# The Effect of Third Dose of Pfizer/BioNTech and Moderna COVID-19 mRNA Vaccines on IgG Antibody Titers

**DOI:** 10.7759/cureus.41696

**Published:** 2023-07-11

**Authors:** Thao A Khuc, Gregery Pequeno, Monica Betancourt-Garcia, Adrienne M Casciato, Marissa Gomez-Martinez, Carlos D Arroyo, Bill D Pope, Shravan Narmala, Sohail Rao

**Affiliations:** 1 Medicine, Doctor's Hospital Renaissance (DHR) Health Institute for Research and Development, Edinburg, USA; 2 Center of Excellence for Trauma Research in the Border Region, Doctor's Hospital Renaissance (DHR) Health Institute for Research and Development, Edinburg, USA; 3 Family Medicine, Doctor's Hospital Renaissance (DHR) Health Institute for Research and Development, Edinburg, USA; 4 Medicine, University of Texas Rio Grande Valley School of Medicine, Edinburg, USA; 5 Hematology and Oncology, Doctor's Hospital Renaissance (DHR) Health, Edinburg, USA

**Keywords:** covid-19, coronavirus disease 2019 (covid-19), who coronavirus, cdc coronavirus, pfizer vaccine, corona virus disease, covid-19 vaccine

## Abstract

With the emergence of severe acute respiratory syndrome coronavirus 2 (SARS-CoV-2) variants, the Centers for Disease Control and Prevention (CDC) authorized the third dose of the Pfizer-BioNTech (BNT162b2) vaccine with the rationale for prolonged elevation of anti-SARS-CoV-2 antibody titers and protection against the SARS-CoV-2 virus.

To better understand how administration of the third dose of the Pfizer/BioNTech coronavirus disease 2019 (COVID-19) vaccine affects the incidence and severity of SARS-CoV-2 infections, we administered the third dose of the Pfizer-BioNTech (BNT162b2) to 189 participants. Blood samples were collected from participants during each of their scheduled visits (baseline, week two, week 12, and week 24) and tested for semi-quantitative anti-SARS-CoV-2 immunoglobulin G (IgG) titers.

Our results showed that administration of the third dose of the Pfizer-BioNTech (BNT162b2) vaccine elicited elevated anti-SARS-CoV-2 IgG antibodies for the 24-week duration of the study. IgG antibody titers were greatest in week two, and progressively decreased by week 12 and week 24, with statistically significant differences between the IgG antibody titers for each collection date.

## Introduction

On August 21, 2021, the United States Food and Drug Administration (FDA) approved the first vaccine Pfizer-BioNTech (BNT162b2) for the protection of individuals aged 12 years and older against coronavirus disease 2019 (COVID-19). The FDA currently recommends a booster vaccine for individuals, “six months or more after the initial vaccination series for individuals - 65 years and older, aged 18+ years who live in long-term care settings, aged 18+ years who have underlying medical conditions, or aged 18+ years who work or live in high-risk settings” [1].

The mechanism of action of messenger RNA (mRNA) vaccines has been well documented to elicit a strong immune response [2]. Injection of the mRNA vaccine triggers dendritic cells to phagocytose the mRNA sequence, ultimately leading to the production of the severe acute respiratory syndrome coronavirus 2 (SARS-CoV-2) spike protein antigens within the cytoplasm of the cell. The release of the spike protein antigens then triggers an immune response in which the antigens are digested by proteasomes for attachment to class one and class two MHC molecules activating the dendritic cells. Activated dendritic cells then travel to lymph nodes where presentation of antigens to T and B cells allows the production of antibodies, proteins that are produced by the immune system as a natural response to a foreign protein or organism [3].

Upon vaccination, we expect there will be an increase of SARS-CoV-2 antibodies in patients’ blood for an estimated 24 weeks (six months) [2]. Previous studies show vaccines elicit an immune response providing lasting immunity that reduces “the risk of infection, seroconversion, and symptomatic illness” [4]. Eventually, a phenomenon known as “threshold of protection” occurs in which the antibodies produced from the vaccines naturally decline to a level that no longer adequately provides protection [5]. B cells localized in lymph nodes also follow this trend, peaking one week after vaccination and slowly declining over a period of six months. During this duration, the B cells are able to efficiently bind to and neutralize the virus [6].

As of November 9, 2022, the increase in SARS-CoV-2 variants has raised increasing concern for another COVID-19 crisis. We hypothesized that subsequent administration of a booster dose of the Pfizer-BioNTech (BNT162b2) vaccine will provide prolonged durations of elevated anti-SARS-CoV-2 antibodies, ultimately leading to enhanced protection from the SARS-CoV-2 virus. With this study, we aimed to determine the anti-SARS-CoV-2 IgG antibody titer levels using a semi-quantitative method at various time points (baseline, week two, week 12, and week 24 after booster).

## Materials and methods

This study was reviewed and approved by Doctor's Hospital Renaissance (DHR) Health Institute for Research and Development Institutional Review Board (IRB). At the time of this study, since boosters were not universally recommended for the general population, authorization from the FDA was requested to administer booster doses to healthcare workers, adults age 65 years or older, or medically immunocompromised patients in the Rio Grande Valley.

Individuals interested in participating in the study received a secure text message from SecureBridge (Prague, Czech Republic: Devart), with a link to complete a screening questionnaire. The questionnaire was conducted on a secure online platform that included screening and demographic questions. Participants of this study were limited to individuals who are at high risk for COVID-19 disease progression, were vaccinated at DHR Health, and have been fully vaccinated for a least six months. Patients were excluded if they had a previous history of allergic reaction to vaccination, active SARS-CoV-2 infection, ≤three weeks of full recovery from SARS-CoV-2 infection, ≤two weeks of any vaccination, and <24 weeks from last dose of Pfizer vaccine. Individuals who met the inclusion criteria were asked to complete a final confirmation screening with a member of the research team and enroll in the study. 

In order to assess the presence of antibodies against SARS-CoV-2, participants were asked to provide a baseline blood sample on the day of administration of the third dose of Pfizer/BioNTech (BNT162b2) vaccine. Blood samples were sent to the laboratory for semi-quantitative anti-SARS-CoV-2 immunoglobulin G (IgG) titers. Subsequently, every study participant received 30 µg in 0.30 mL of Pfizer/BioNTech (BNT162b2) administered intramuscularly in the deltoid. Participants were monitored for 15 minutes post-vaccination before being released.

Patients were scheduled to come back for a blood draw at week two, week 12, and week 24 after their booster dose to check for SARS-CoV-2 IgG antibodies. At the week 24 visit, or over the phone, patients were asked the below questionnaire (Table 1).

**Table 1:** Week 24 visit questionnaire. Y: yes; N: no; COVID-19: coronavirus disease 2019

Question	Y or N	If Y, explain
Did you receive the primary immunizations and if yes, what type of vaccination?		
Did you ever test positive for COVID-19 and if yes, were you hospitalized?		
Did you receive a convalescent plasma infusion?		
Did you receive monoclonal antibody infusion?		
Did you receive Remdesivir, and if yes was it a five-day or 10-day course?		
Did you receive ivermectin?		
Did you receive or take anything else to fight or prevent a COVID-19 infection?		
Do you have any current health complications?		
Did you receive any additional COVID-19 vaccine?		
Did you have any adverse reaction to COVID-19 vaccine administration?		

All antibody titer results included in this study were processed at a laboratory following the semi-quantitative anti-SARS-CoV-2 IgG two-step enzyme immunoassay protocol described in more detail in the figures in Appendices. Categorical data were summarized using their respective percentages and frequencies. Numerical data were summarized using the minimum, maximum, median, first and third quartiles, mean, and standard deviation. The IgG antibody level was used as the outcome variable and analyzed using linear regression. Univariate regression analyses were conducted for patients' age, gender, and primary immunization. Variables were tested for equal variances using the Brown-Forsythe test. When comparing antibody levels between populations in categorical variables, means were compared using a pooled t-test if the variances were equal and Welch’s t-test if the variances were unequal. To analyze the IgG antibody levels at the different time points, the median antibody levels were calculated and compared using the Wilcoxon signed-rank test. All statistical analyses were conducted using JMP 16.1.0 (Cary, NC: SAS Institute Inc.). Statistical testing was two-sided and performed at a significance (α) level of 0.05.

## Results

As shown in Table 2, a total of 189 participants were enrolled in the study during the recruitment phase. Of these, the participants that failed to complete follow-up visits (n=109) or got their fourth dose before completion of the study (n=3) were excluded, leaving 77 participants for analysis. Of the 77 participants, 46 were female (59.70%), and the age range of participants fell between 22 and 81 years with an average age of 51 years (Table 3).

**Table 2:** Participant enrollment and follow-up visits. As the study progressed, participants were considered lost to follow-up if they missed any of their follow-up visits.

Variables	Baseline (week 0)	Visit one (week two)	Visit two (week 12)	Visit three (week 24)
Enrolled	189	189	163	135
Lost to follow-up	0	26	28	58
Total	189	163	135	77

**Table 3:** Demographics of the study participants. The majority of the patient population were between 44 and 60 years of age, females, and/or who received the Pfizer vaccine.

Sociodemographic characteristics		N	%
Age (years)	8-25	3	3.90
	26-43	22	28.57
	44-60	29	37.66
	61-77	22	28.57
	78+	1	1.30
Gender	Female	45	58.44
	Male	32	41.56
Primary immunization	Pfizer	70	90.90
	Moderna	7	9.10

The participants’ SARS-CoV-2 IgG antibody results were collected at baseline, week two, week 12, and week 24 after administration of the vaccine. There was an average of 223 days from the administration of the second dose to the administration of the third dose. Participants were allowed to complete their two-week follow-up visit from day 14 to day 28. Participants scheduled for the week 12 visit came in for their follow-up between 53 and 110 days after the third dose of vaccine administration. The final 24-week follow-up visit was conducted from day 121 to 225 days after the third dose.

For the baseline blood draw, the average antibody titer was 39.34 AU/mL (SD=105.22). After analyzing the 25th percentile (Q1), the result was 10 AU/mL, and the median number of antibodies was 16.92 AU/mL. Additionally, for the 75th percentile (Q3), the results were 32.76 AU/mL. The minimum result of IgG titers calculated was 10 AU/mL and the maximum number was 884 AU/mL.

Two weeks after the administration of the third dose, the mean number of antibody titers increased to 293.35 AU/mL (SD=331.93, p<0.0001). The Q1 results were found to be 132.19 AU/mL and the median value was 198.03 AU/mL. The Q3 recorded value was 313.02 AU/mL. The lowest and highest antibody titer found in participants was 52.92 AU/mL and 1960 AU/mL, respectively.

**Figure 1:**
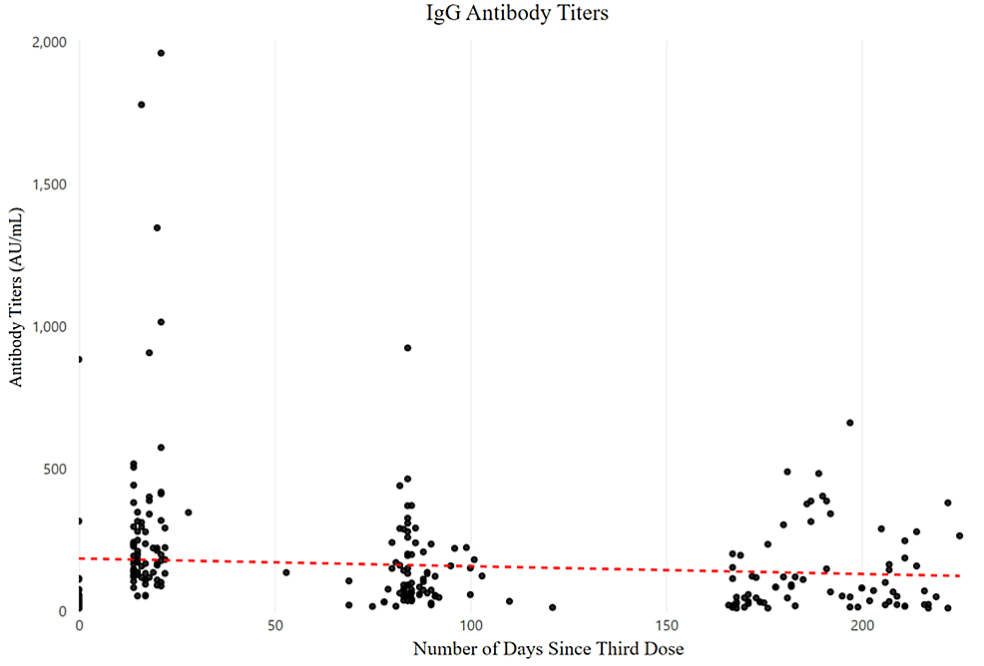
The effect of elapsed days since the third dose on immunoglobulin G (IgG) antibody titers.

As shown in Figure 1, the average antibody titer at week 12 was 143.91 AU/mL (SD=137.69, p<0.0001). The first quartile range (Q1) presented a number of 55.44 AU/mL and a median of 98.20 AU/mL, while the third quartile range (Q3) was 199.22 AU/mL. The lowest number of IgG titers for the results was 15.95 AU/mL, and the most elevated number of IgG titers was 924 AU/mL.

The average antibody titer was 127.04 AU/mL (SD=139.94, p=0.0075) 24 weeks after the third dose. The Q1 value was 25.35 AU/mL and the median was 70.49 AU/mL, whereas the Q3 was 174.82 AU/mL. The least amount of IgG antibody titers for this time point was 10 AU/mL, and the highest was 661 AU/mL.

## Discussion

Third dose administration of the Pfizer/BioNTech (BNT162b2) vaccine-induced elevation of IgG antibodies for the 24-week duration of the study. IgG antibody levels were elevated at each collection date (week two, week 12, and week 24) and progressively declined thereafter. Differences in IgG antibody levels at each date were statistically significant with mean values of 293.35 (week two), 143.91 (week 12), and 127.04 (week 24). Persistent elevation of IgG antibodies from baseline to week 24 shows that the third dose of vaccine administration is effective in evoking an immunological response for that duration [7]. These results support previous studies that highlight the effects the third dose vaccine had on enhancing the immune response to SARS-CoV-2 infections [8]. Most notable of these studies was a study in Israel that included 1,137,804 adults aged 60 years and older who received the first two Pfizer/BioNTech (BNT162b2) vaccines five months prior to the third dose [9].

Several limitations are noteworthy in this study. The sample selected for this study were either healthcare workers, adults aged 65 years or older, or patients that were medically considered immunocompromised; thus, results obtained from this study may not be applicable to populations outside the selected population.

Financial limitations also influenced the number of patients that returned for follow-ups. The study relied on a COVID-19 HRSA grant, which covered any expenses related to the study. Upon depletion of the grant, patients were responsible for covering fees related to the study, which prompted many to withdraw from the study. Financial limitations also restricted the ability to test for both neutralizing antibody tests and cell-mediated immunity testing. Therefore, it was not possible to determine the level of involvement of IgG antibody response or cellular-mediated immunity in the incidence and severity of SARS-CoV-2 infections. IgG antibody titers can be influenced by a variety of factors, such as previous infections with SARS-CoV-2 and patient exposure to monoclonal antibody (MAb) infusion, potentially explaining why some patients had higher levels than the average upon follow-up visits [2].

## Conclusions

Our results show that IgG titers remain sustained for an average of 12 weeks, with peak levels during week two, before a significant decrease as measured at 24 weeks. These results indicate that over time, subsequent booster shots should be provided to promote continuous elevated IgG antibody titers for protection against the SARS-CoV-2 virus. Regarding immunity provided between the Pfizer and Moderna vaccines, there was no significant difference in immunity provided post-vaccination.

## Figures and Tables

**Figure 2:**
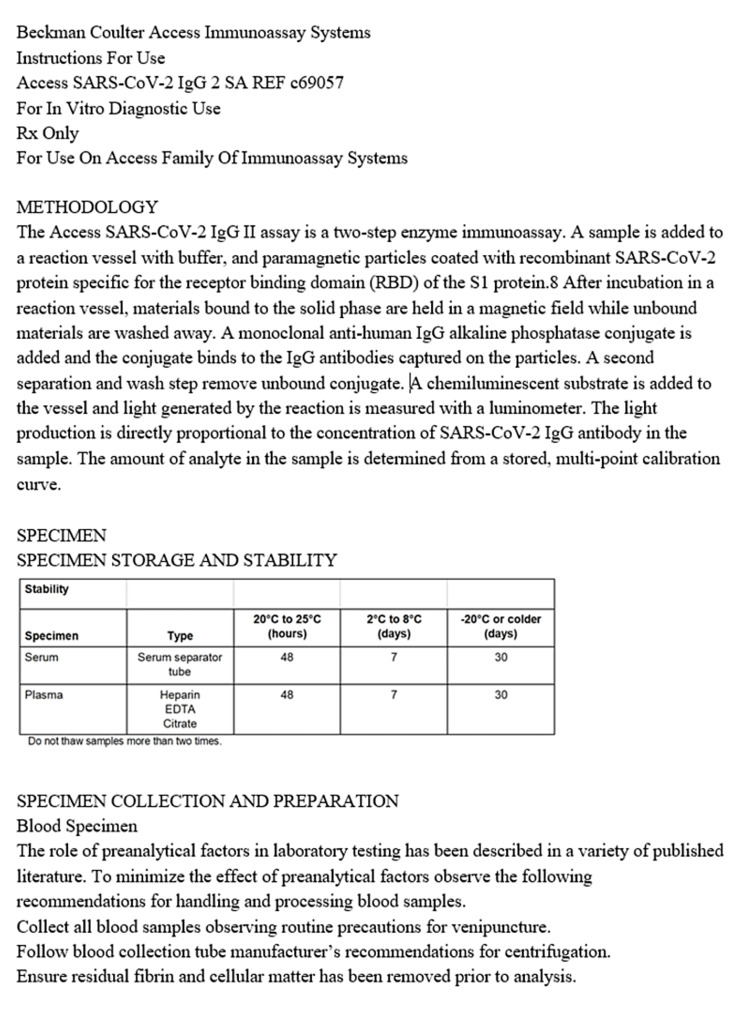
Beckman Coulter Access Immunoassay Systems instructions for use (page 1 of 3).

**Figure 3:**
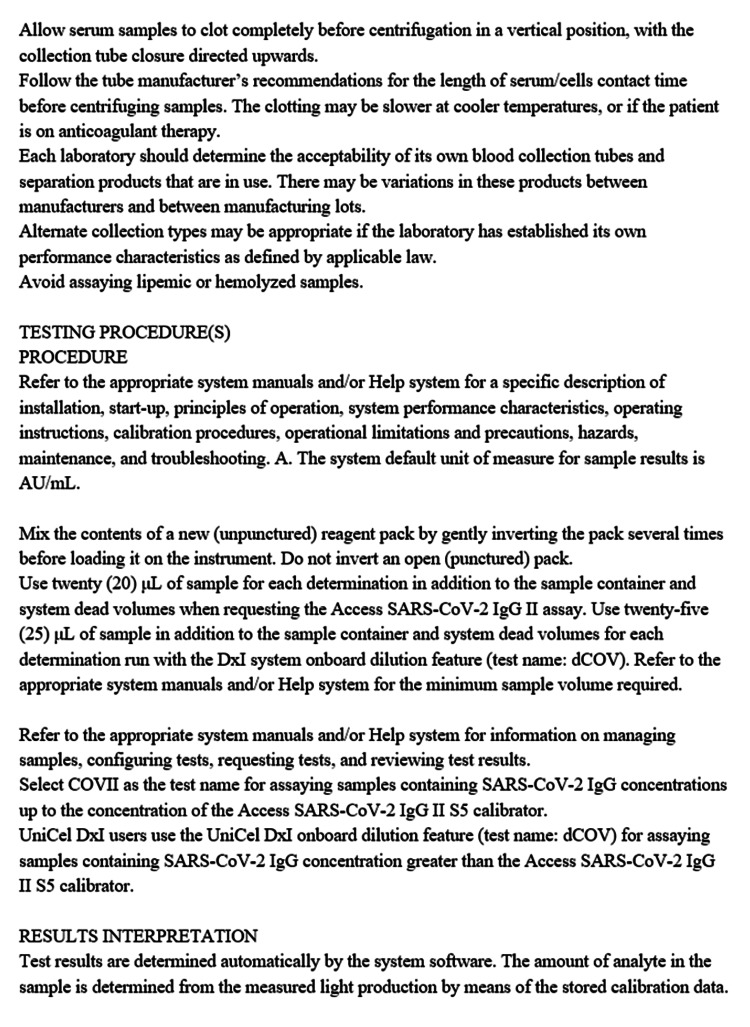
Beckman Coulter Access Immunoassay Systems instructions for use (page 2 of 3).

**Figure 4:**
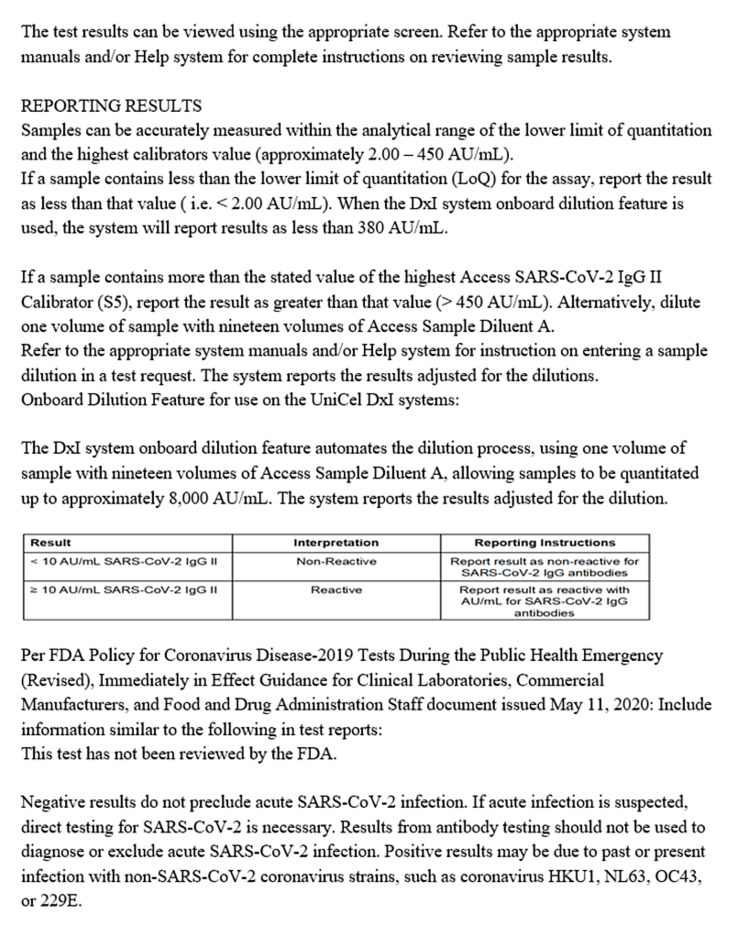
Beckman Coulter Access Immunoassay Systems instructions for use (page 3 of 3).
